# Evaluation of hematological parameters as an indicator of disease severity in Covid‐19 patients: Pakistan's experience

**DOI:** 10.1002/jcla.23809

**Published:** 2021-05-24

**Authors:** Abdul Waris, Misbahud Din, Atiqa Khalid, Raees Abbas Lail, Asmat Shaheen, Nida Khan, Mehboob Nawaz, Abdul Baset, Imtiaz Ahmad, Muhammad Ali

**Affiliations:** ^1^ Department of Biotechnology Quaid‐i‐Azam University Islamabad Islamabad Pakistan; ^2^ Department of Pathology Sahiwal Medical College, Affiliated with University of Health Sciences Lahore Lahore Pakistan; ^3^ Department of Zoology Bacha Khan University Charsadda Charsadda Pakistan; ^4^ Department of Botany Bacha Khan University Charsadda Charsadda Pakistan; ^5^ KMU‐Institute of Medical Sciences Kohat Kohat Pakistan; ^6^ Department of Obstetrics and Gynecology Hayat Medical Complex Peshawar Peshawar Pakistan

**Keywords:** biomarkers, COVID‐19, disease severity, hematological parameters

## Abstract

**Background:**

The severity of COVID‐19 could be evaluated by examining several blood parameters mainly white blood cell (WBC) count, granulocytes, platelet, and novel hemocytometric markers neutrophils to lymphocyte ratio (NLR), platelet‐to‐lymphocyte (PLR), and lymphocyte to monocyte ratio (LMR). The current study was conducted to investigate alteration in blood parameters and their association with the severity and mortality of COVID‐19 patients.

**Methodology:**

An observational cross‐sectional study was conducted retrospectively, a total of 101 COVID‐19 positive patients were examined: 52 were mild, 24 were moderate, 09 were severe, and 16 were critically diseased patients. We also recorded 16 deaths associated with the critical group. The overall mean age observed in our study was 48.94 years, where the mean age for critical individuals was 62.12 ± 14.35 years.

**Results:**

A significant association between the disease severity and elevation in blood parameters were observed. The WBC's and granulocyte count were significantly increased (*p* value <0.001) while the mean platelet count (165.0 × 10^9^/L) and red blood cell volume distribution width (RDW) were decreased in the critical group (57.86%) compared to mild group's patients (177.3%) (*p* = 0.83). The lymphocytes count was decreased in critical patients (1.40 × 10^9^/L) compared to mild patients (1.92 × 10^9^/L) (*p* = 0.28). A significant association was observed in platelet‐lymphocyte ratio (*p* < 0.001), Neutrophil‐Lymphocyte ratio (*p* = <0.001), and Lymphocyte‐Monocyte ratio (0.011).

**Conclusion:**

These blood parameters could be used as a suitable biomarker for the prognosis and severity of COVID‐19. Evaluating novel hemograms NLR, PLR, and LMR can aid clinicians to identify potentially severe cases at early stages, initiate effective management in time, and conduct early triage which may reduce the overall mortality of COVID‐19 patients.

## Graphical abstract

Hematological findings as indicator of disease severity in Covid‐19 patients.

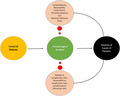

## 1 INTRODUCTION

The coronavirus disease 2019 (COVID‐19) caused by Severe Acute Respiratory Syndrome Corona Virus 2 (SARS‐CoV‐2) was first described in the late December 2019 in Wuhan, China.^1^, ^2^ The COVID‐19 is a high contagious disease and spread around the globe within a short time, and the world health organization (WHO) has declared it a pandemic on March 12, 2020.^3^

COVID‐19 has varied clinical manifestations, ranging from mild flu‐like symptoms and asymptomatic phase to life‐threatening acute respiratory distress syndrome (ARDs), and organ failure that may lead to death.^4^ The initial symptoms of COVID‐19 are shortness of breath, cough, fatigue, fever, dyspnea, myalgia, and muscle pain. However, they could progress to acute respiratory distress syndrome (ARDS), multi‐organ dysfunction, shock, and metabolic acidosis when the condition becomes worsened.^5^ To date, COVID‐19 has affected 212 countries/regions around the world. As of 16 April 2021, the WHO reported approximately 138,688,383 confirmed cases with 2.9 million mortalities globally.^6^ Approximately, 50% of COVID‐19 patients were asymptomatic carriers and presymptomatic. And close contact tracing and continuous surveillance could help detect these presymptomatic patients and carriers. At the start of an infection and in the incubation period, there are mild flu‐like symptoms. In fact, nearly 2 or 3 of every four subjects that have positive real‐time Reverse transcriptase PCR throat swab results remain asymptomatic and only 10% of the symptomatic patients develop interstitial pneumonia, shortness of breath, acute respiratory distress syndrome either with, or without sepsis and multi‐organ failure.^7^ According to the National Health Commission of China, the protocol published (version 7) for diagnosis and treatment for COVID‐19, the COVID‐19 severity is classified into four levels based on the clinical manifestations: critical, severe, moderate, and mild disease.^8^ Different respiratory factors such as oxygen saturation rate, lesion progression in pulmonary rate, and respiratory rate were the key factors considered during the classification of the severity levels of COVID‐19.^9^ Patients with severe COVID‐19, mostly critical individuals, usually have dysfunction complications of other organs, such as shock, septic, disseminated intravascular coagulation (DIC), and heart failure. In critical cases, some thrombotic complications such as strokes, venous thromboembolism, and ischemic limbs have been reported.^10^

Except for clinical symptoms and pulmonary computed tomography (CT) findings, most confirmed COVID‐19 patients revealed laboratory fluctuations in different serological parameters, including renal and liver function tests, coagulation parameters, and inflammatory, biochemical and hemocytometric parameters.^9^, ^11^, ^12^ To show the prognosis and hyperinflammation state, a combination of laboratory tests has been evaluated. The combination of the various test includes platelet‐to‐lymphocyte (PLR) and neutrophils to lymphocyte ratio (NLR). COVID‐19 leads to variation in the hematological parameters, including lymphocytes, white blood cells, platelets, neutrophils, etc.^12^, ^13^, ^14^ These variations are different from case to case and level of the disease severity. Lymphopenia has been previously reported in about 35%–85% of patients and was the most common blood count abnormality.^10^, ^11^, ^12^ Moreover, knowledge about the risk of infections along with other comorbid conditions would provide valuable insights on risk stratification and making a clinical decision in severe COVID‐19 patients.^15^ According to different studies, NLR in severe patients was raised as compared to those with mild or moderate disease.^4^, ^16^ Further, Liu et al^16^ found out in hospitalized patients that NLR serves as an independent risk factor for mortality. CBCs are the most appropriate and potent laboratory examination. The main objective of this research was to evaluate and review the variations among CBC level of COVID‐19 patients with the disease severity level and how the CBC level changes after the onset of disease to identify the stage and disease's key indicator to provide information for the diagnosis and treatment basis for health professionals. Moreover, this study aimed to explore novel inflammatory markers NLR, PLR as a valuable marker in predicting severity and outcome of COVID‐19 infection as they were previously reported in different infections and inflammatory conditions.^17^

## 2 MATERIALS AND METHODS

### 2.1 Study design

A retrospective cross‐sectional study was conducted from May 2020 to September 2020 in the Pathology Department of Sahiwal Medical College, Sahiwal.

### 2.2 Patients

Our study participants include *n* = 101 patients who were tested positive for SARS‐CoV‐2 through real‐time reverse transcriptase PCR and admitted in various isolation wards of District Head Quarter Hospital (DHQ) Sahiwal. Among them, 60 were males individuals while 40 were females. The overall average age was 15–85 years. The demographic information such as gender, age, and co‐morbidities was also recorded from each individual as shown in Tables 1 and 2.

**TABLE 1:** Demographic information of various disease groups

Demographics	Total	Mild	Moderate	Severe	Critical
	*N* = 101	*N* = 52	*N* = 24	*N* = 09	*N* = 16
Age	48.9	43.24	49.1	56.6	62.1
Gender					
Male	69 (68.3%)	39 (38.6%)	14 (13.6%)	6 (5.94%)	11 (10.89%)
Female	32 (31.7%)	13 (12%)	10 (9.9%)	3 (2.9%)	5 (4.95%)

**TABLE 2:** Comorbidities in COVID‐19 patients and their association with disease severity

Comorbidities	Total	Mild	Moderate	Severe	Critical	*p* Value^a^
	*n* (%)	*n* (%)	*n* (%)	*n* (%)	*n* (%)	
Hypertension	52 (51.4%)	30 (57.6%)	11 (21.1%)	3 (5.7%)	8 (15.3%)	0.003
Diabetes mellitus	51 (50.4%)	27 (52.9%)	11 (21.5%)	3 (5.8%)	10 (19.6%)	
Liver diseases (HCV +vet, HBV +vet, CLD)	11(0.8%)	5 (45.4%)	3(27.7%)	1(9.09%)	2(18.1%)	
Ischemic heart disease	8(7.9%)	3 (37.5%)	2 (25%)	1(12.5%)	2(25%)	
Asthma	7(6.9%)	3 (42.8%)	1(14.2%)	1 (14.2%)	2 (28.5%)	

^a^ Chi‐Square test.

### 2.3 Sample processing procedure

About 3 ml of venous blood was collected on the second day of admission to the hospital using the EDTA vacutainer tube. The blood samples collected from each individual were analyzed for complete blood count (CBC) examination using Automatic Hematology Analyzer Swelab Alfa Standard (Boule Medical AB). The blood routine indicators included lymphocyte count (LYM), lymphocyte %, platelet count (PLT), white blood cell count (WBC), platelet volume distribution width (PDW), PDW%, red blood cell count (RBC), red blood cell volume distribution width (RDW), red blood cell volume distribution width (RDW), hematocrit (HCT), mean corpuscular volume (MCV), mean corpuscular‐hemoglobin (MCH), mean corpuscular‐hemoglobin concentration (MCHC), mean platelet volume (MPV), hemoglobin (HGB), PLT‐I, granulocytes count, Granulocyte ratio (G%), neutrophil ratio, and neutrophil count.

### 2.4 Exclusion and inclusion criteria

All those individuals who were tested positive for the SARS‐CoV‐2 according to the WHO and CDC guidelines for the detection and diagnosis of COVID‐19 were included in this study. Individuals below 18 years of age and those with missing data were excluded.

### 2.5 Definitions

According to the National Health Commission China guidelines (trial version 7) for novel coronavirus pneumonia diagnosis and treatment protocol, COVID‐19 patients have been classified into different groups, that is, critical, severe, moderate, and mild, based on the severity of the disease. All the patients will have a critical disease that meets any of the following clinical manifestations and problems: failure of the organ(s) that requires intensive care unit (ICU) for monitoring and treatment. Severe disease is defined as patients having 935 of saturation rate or less at rest, per min breaths is ≥30, over 24–48 h, >50% lesion progression in pulmonary imaging, the arterial pressure of oxygen (PaO2)/fractional concentration of oxygen (FiO2) is ≤40kpa. While the disease has respiratory symptoms and radiological findings of pneumonia and fever but without various critical and severe features, it was termed as moderate. The mild disease level is defined as patients with slight clinical manifestations but no appearance of pneumonia on radiological imaging. This study has been approved by the Ethical Review Board of the Sahiwal Medical College, Punjab province, Pakistan.

### 2.6 Neutrophil‐to‐lymphocyte ratio

**FIGURE 1:**
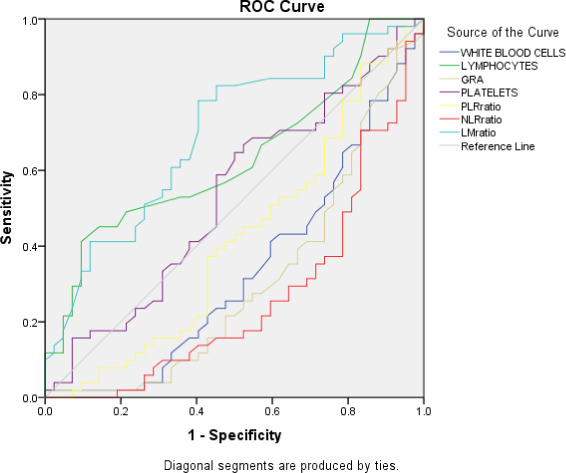
ROC curves reveal the relative diagnostic performance of haematological parameters and age, forecasting severity of disease in patients with SARS‐CoV‐2 infection, white blood cell count (WBC), neutrophils to lymphocyte ratio (NLR), platelet‐to‐lymphocyte (PLR), and lymphocyte to monocyte ratio (LMR)

Neutrophil‐to‐lymphocyte ratio is determined by dividing the relative percentage of neutrophils by lymphocytes. In normal individuals, it should be <3 but a ratio of >3 signifies acute infection, and a ratio of more than nine reveals sepsis. But when it comes to the cut‐off value, there is a lot of diversity of NLR in populations, with some studies suggesting a cut‐off value of 4.^18^ The NLR of our patients was also analyzed as shown in Table 3 and Figure 1. There are many reports in the literature that concluded there is an association between inflammatory condition and NLR.^19^ Simona et al^20^ identified NLR as a predictor of neurological deterioration following acute cerebral hemorrhage and can help in the risk stratification of patients. Additionally, in healthy subjects, raised NLR may be indicative of underlying deranged glucose metabolism. It can also be considered as a marker of glucose control in type 2 diabetics in addition to HbA1c.^21^ Further, Milena et al found it as an independent predictor of symptomatic Hemorrhagic transformation (sHT), which is a complication of acute ischemic stroke (AIS). Interestingly, the study shows that systolic blood pressure and NLR were independently associated with sHT. Hence, NLR at admission can accurately predict sHT in patients with AIS undergoing revascularization.^22^ It can also as a diagnostic tool in inflammatory bowel disease.^23^ This ratio has also been considered as a determinant of outcomes of various malignancies and coronary arterial diseases as well as patients suffering from recurrent ulcerative colitis. All these conditions are associated with overt inflammation as in COVID‐19.

**TABLE 3:** Hematological parameters in various disease group of COVID‐19 patients

	Total	Mild disease	Moderate disease	Severe	Critical	*p* Values
	(*n* = 101)	*n* = 52	*n* = 24	*n* = 9	*n* = 16	
WBC 10^9^/L	8.00 (7.14–8.86)	6.76 (5.79–7.73)	8.25 (6.55–9.95)	7.77 (4.57–10.97)	11.79 (8.85–14.72)	<0.001
Lymph 10^9^/L	1.69 (1.53–1.85)	1.92 (1.67–2.17)	1.42 (1.21–1.63)	1.58 (0.94–2.23)	1.40 (0.99–1.81)	0.28
MID %	0.335 (0.28–0.38)	0.32 (0.25–0.39)	0.30 (0.22–0.37)	0.32 (0.19–0.44)	0.45 (0.30–0.59)	0.25
Granulocytes 10^9^/L	5.52 (4.79–6.25)	4.44 (3.64–5.24)	6.09 (4.78–7.40)	5.86 (2.68–9.04)	8.78 (5.66–11.89)	<0.001
HGB g/dl	12.93 (12.6–13.26	12.95 (12.47–13.42)	12.66 (11.91–13.41)	13.13 (11.78–14.48)	13.18 (12.38–13.98)	0.77
MCH Pg	27.22 (26.50–27.94)	26.77 (25.99–27.50)	26.60 (25.56–27.60)	28.36 (25.72–31.00)	28.97 (25.56–32.38)	0.10
MCHC g/dl	32.00 (31.61–32.39)	32.29 (31.95–32.63)	31.4 (30.41–30.40)	31.7 (30.71–32.68)	32.13 (30.40–33.87)	0.31
RBCs 10^12^/L	4.94 (4.74–5.15)	4.95 (4.69–5.21)	4.97 (4.46–5.48)	5.10 (3.73–6.48)	4.80 (4.53–5.08)	0.91
MCV fl	83.54 (81.79–85.29)	82.63 (80.63–84.63)	83.65 (80.32–86.98)	86.45 (72.67–100.23)	84.69 (80.21–89.17)	0.62
HCT %	40.70 (39.34–42.06)	40.39 (38.81–41.96)	39.85 (37.38–42.31)	39.56 (35.74–33.38)	43.60 (37.56–49.65)	0.31
RDW %	121.08 (−242.96)	177.30 (−416.80)	60.61 (54.41–66.81)	69.07 (59.76–78.38)	57.86 (49.66–66.06)	0.83
PLTs 10^9^/L	209.23 (191.83–226.62)	217.03 (191.52–240.55)	223.73 (185.93–261.54)	205.55 (120.38–290.72)	165.06 (123.44–206.67)	0.16
MPV fl	10.0 (8.47–11.53)	9.02 (8.60–9.43)	12.60 (5.93–19.26)	9.34 (8.62–10.06)	9.74 (9.15–10.33)	0.31
PDW fl	12.70 (12.03–13.38)	12.18 (11.81–12.56)	13.70 (10.92–16.48)	12.53 (11.31–13.75)	12.21 (12.05–14.37)	0.31
PCT %	0.19 (0.17−0.20)	0.19 (0.17–0.21)	0.19 (0.15–0.23)	0.19 (0.12–0.25)	0.16 (0.11–0.20)	0.51
P‐LCR %	24.17 922.65–25.70)	23.08 (21.28–24.87)	25.40 (21.12–29.68)	23.52 918.36–28.68)	26.49 (22.29–30.69)	0.39
P‐LCC %	45.78 (41.71–49.86)	45.49 (41.28–49.69)	48.40 (35.41–61.40)	46.77 (29.60–63.94)	41.50 (30.66–52.55)	0.81
PLR (Platelet‐to‐Lymphocyte ratio)	160.6 (128.3–193.01)	131.5 (112.2–150.8)	180.6 (138.1–223.2)	139.5 (82.7–196.2)	238.3 (44.96–431.7)	<0.001
NLR (Neutrophils‐to‐Lymphocyte ratio)	3.99 (3.32–4.67)	2.70 (2.12–3.29)	4.95 (3.70–6.21)	5.11 (1.90–8.32)	6.87 (3.68–10.07)	<0.001
LMR (Lymphocyte‐to‐Monocyte ratio)	6.96 (6.01–7.90)	8.32 (6.88–9.75)	5.74 (4.38–7.09)	5.54 (3.02–8.07)	4.37 (1.98–6.75)	0.011

Abbreviations: HCT, hematocrit; HGB, hemoglobin; MCH, mean corpuscular‐hemoglobin; MCHC, mean corpuscular‐hemoglobin concentration; MCV, mean corpuscular volume; MPV, mean platelet volume; PDW, platelet volume distribution width; PLTs, platelet count; RBCs, red blood cells; RDW, red blood cell volume distribution width; WBC, white blood cell.

### 2.7 Lymphocyte to monocyte ratio

Dividing absolute lymphocyte count (×10^9^ cells/µl) with absolute monocyte count (×10^9^ cells/µl) gives this ratio.^24^ The normal range is 3–9, with diversity amongst populations.^25^ Decreased lymphocytes and elevated monocytes result in declined lymphocyte‐to‐monocyte ratio among ICU or deceased patients, thus rendering decreased ratio of lymphocyte‐to‐monocyte as an indicator of poor prognosis increased chances of mortality among patients suffering from COVID‐19 correlating with few studies. The mean value of the lymphocyte‐to‐monocyte ratio coincides with the mean of another study calculating LMR in coronavirus disease patients.^12^

### 2.8 Platelet‐to‐lymphocyte ratio

Absolute platelet counts (×10^9^ cells/µl) hence divided by absolute lymphocyte count (×10^9^ cells/µl), gives this ratio and it usually 50–150 but it also has a great diversity amongst people.^7^ In this study, raised platelet count (thrombocytosis) was determined in COVID‐19 patients at the time of initial phase of infection (admission) and critical phase of infection (intensive care unit) and death as compared with patients in isolation wards or recovered in parallel with outcomes of recent studies. Alternatively, a decrease in lymphocyte count (lymphocytopenia) was detected in critical phase patients, with predominantly elevated levels of platelet‐to‐lymphocyte ratio among patients under treatment in ICU or declined in comparable with those in their initial stage of disease (admission) or admitted in isolation wards. Thus, supporting the evidence of platelet‐to‐lymphocyte ratio as one of the independent mediator predicting mortality and prognosis in critically ill sufferers synchronizing with the results of another study.^26^, ^27^ The means of platelet‐to‐lymphocyte ratio detected among patients under treatment in the isolation ward and intensive care units coincide with the means of the study mentioned above Moreover, As noted by Xu et al,^28^ in acute ischemic stroke patients who are treated with intravenous thrombolysis, elevated PLR levels seem to be independently associated with an unfavorable outcome and death after three months. PLR is also identified as a diagnostic tool in inflammatory bowel syndrome.^23^ Similarly, Mean Platelet volume to lymphocyte ratio (MPVLR) was also found to be associated with frailty in type 2 diabetic individuals.^23^

### 2.9 Statistical analysis

Different statistical tools were used for the analysis of data. The continuous variables were demonstrated as standard deviation (SD) and mean or interquartile (IQR), and median. t Test and Mann–Whitney *U* test were used to analyze normally distributed non‐continuous variables and abnormally distributed continuous variables, respectively. Different categorical variables were expresses as percentage and frequency rate. Fisher's exact test and χ^2^ test were used for the analysis of categorical variables. Various comparisons among different groups were also performed using the Bonferroni adjustment method. *p*‐value of ≤0.05 was considered statistically significant. Statistical Package for the Social Sciences (SPSS) (version 23.0) was used for all the statistical analyses.

## 3 RESULTS

A total of 101 COVID‐19 positive individuals were examined in this study. The patients were previously screened through RT‐PCR for the SARS‐CoV‐2 infection and were found positive for the viral infection. Among the total individuals (*n* = 101), 69 (68.3%) were male individuals while 32 (31.7%) were female individuals. Male individuals were more affected than female individuals. The severe and critical diseased patients were mostly older individuals compared with mild and moderate groups. The overall mean age was 48.94 years where the mean age for critical individuals was 62.12 ± 14.35 years. Comorbidities were also mentioned for also each group and a statistically significant difference was observed between mortality and disease severity as shown in Table 2.

**FIGURE 2:**
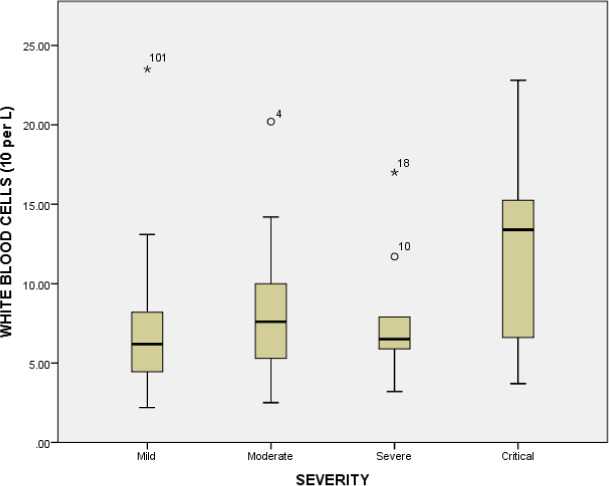
White blood cell counts (10^9^ cells per L) in various diseased groups. Chi‐square test was applied and p < 0.05 was considered as significant at 95% confidence of interval.

**FIGURE 3:**
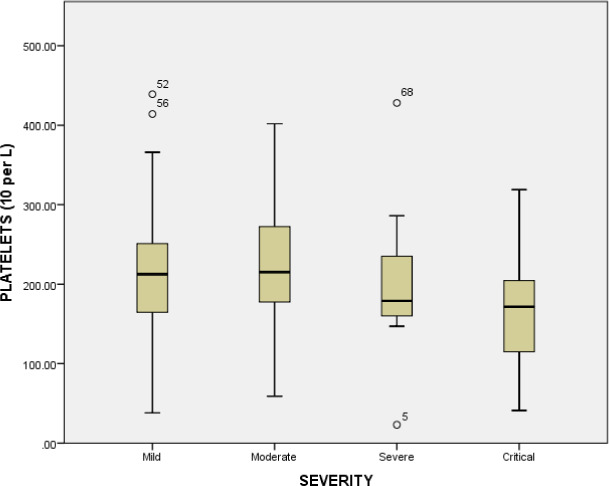
Platelets count (10^9^ cells per L) in various diseased groups. Chi‐square test was applied and p < 0.05 was considered as significant at 95% confidence of interval.

**FIGURE 4:**
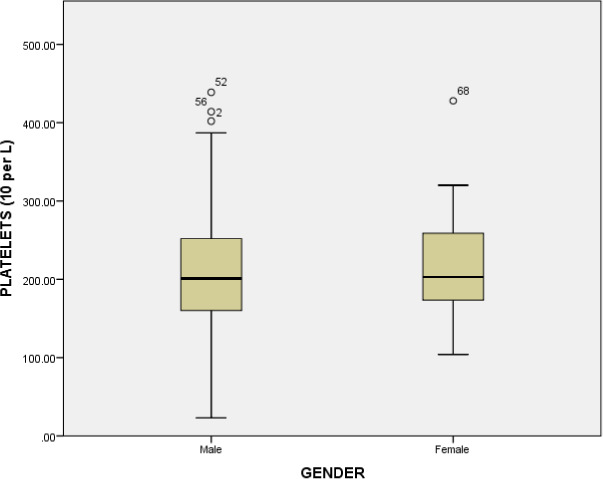
Association of platelet count (10^9^ cells per L) with gender. Chi‐square test was applied and p < 0.05 was considered as significant at 95% confidence of interval.

**FIGURE 5:**
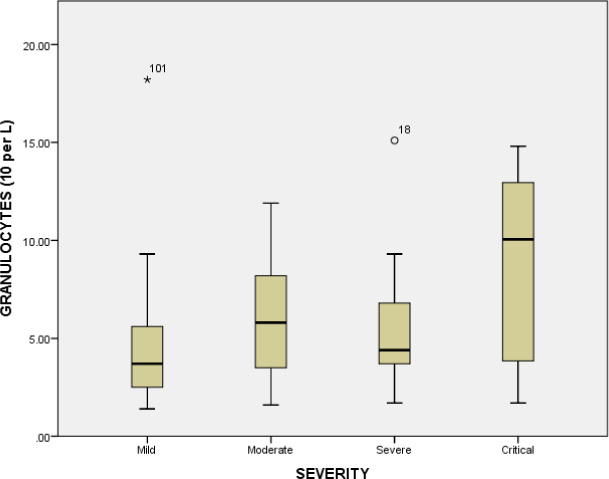
Granulocytes level (10^9^ cells per L) in various diseased groups. Chi‐square test was applied and *p* < 0.05 was considered as significant at 95% confidence interval. The critically diseased individuals represented the highest level of granulocytes as compared to other diseased groups

The hematological parameters such as white blood cells, lymphocyte count, granulocytes, hemoglobin, mean corpuscular‐hemoglobin, mean corpuscular‐hemoglobin concentration, red blood cells, mean corpuscular volume, hematocrit, red cell distribution width, percentage of red cell distribution width, platelets, mean platelet volume, platelet distribution width, platelet count, platelet large cell ratio were examined across the comparative groups. The white blood cells were significantly increased (*p* value = <0.001) in critical patients compared to mild individuals (6.76 × 10^9^/L). Similarly, significant difference (*p* value = <0.001) of white blood cells was observed between the moderate patients (8.25 × 10^9^/L) and severe patients (7.77 × 10^9^/L), and between moderate (8.25 × 10^9^/L) and critical patients (11.79 × 10^9^/L) (*p* value = 0.039). The white blood cell count was significantly increased in critical patients represented in Figure 2. Similarly, the mean platelets count was significantly decreased in critical group (165.0 × 10^9^/L) compared to moderate group (223.73 × 10^9^/L), mild group (217.03 × 10^9^/L) and severe group (205.55 × 10^9^/L) (*p* value = 0.16). The platelet count (10^9^ cells per L) in various groups is represented in Figure 3. A significant decrease was observed in critical disease patients. The association of platelet count with gender is represented in Figure 4. No significant association between gender and platelet count elevation was observed in COVID‐19 patients. The red blood cell count was increased in the severe group (5.1 × 10^12^/L); however, it was similar in other groups (mild group 4.95 × 10^12^/L, moderate group 4.97 × 10^12^/L) (*p* value = 0.91). The red blood cells count in critical patients was 4.80 × 10^12^/L. The granulocytes were significantly increased in critical patients (8.78 × 10^9^/L) compared to severe (5.86 × 10^9^/L) and moderate groups (6.09 × 10^9^/L) (*p* value = <0.001). The critical diseased individuals represented the highest value of granulocytes compared to other diseased groups (Table 3 and Figure 5).

The red cells distribution width was significantly decreased (57.86%) in critical patients compared to mild patients (177.3%) (*p* value = 0.83). The lymphocytes count was decreased in critical patients (1.40 × 10^9^/L) compared to mild patients (1.92 × 10^9^/L) (*p* value = 0.28). A small difference in the hemoglobin level was observed which indicates no significant association (0.77 g/dl) with the severity of disease (*p* value = 0.77). The mean corpuscular hemoglobin was also observed similar across the different comparative groups (*p* value = 0.1). Similarly, the mean corpuscular‐hemoglobin concentration was also not elevated with the severity of the disease (*p* value = 0.31). The mean corpuscular volume was similar between mild patients (82.63 fl) and moderate patients (83.65 fl) however a little increase was observed in severely diseased patients (86.45 fl). The platelet large cell ratio was increased in critical patients (26.49%) compared to severe patients. Similarly, the platelet large cell count was decreased in critical patients (41.50%) compared to severe patients. However, no significant association was observed in both parameters with severity of the disease.

The PLR was significantly increased in the critical group of patients (238.3) as compared to severe (139.5), moderate (180.6), and mild (131.5) disease groups. A significant association (*p* value = <0.001) was observed in critical disease group patients and various other disease group groups as shown in Table 3. A significant increase was also observed in NLR in various disease groups. The highest value of NLR was recorded in critically ill patients (6.87) followed by severe, moderate, and mild disease groups. A significant association (*p* value = <0.001) was observed in various disease groups as shown in Table 3. Similarly, a decrease in the LMR was observed in the critical disease group patients compared to other groups. The lowest value of LMR was recorded for critically ill patients (4.37). A significant association (*p* value = 0.011) was observed in the critical disease group as compared to other groups. ROC curves reveal the relative diagnostic performance of hematological parameters and age, forecasting the severity of disease in patients with SARS‐CoV‐2 infection, WBC, white blood cell; NLR, neutrophil‐to‐lymphocyte ratio; PLR, platelet‐to‐lymphocyte ratio; LMR, lymphocyte‐to‐monocyte ratio as shown in Figure 1.

## 4 DISCUSSION

The second wave of COVID‐19 has been started and cases are steadily increasing around the globe. There is a need to assess the disease severity and mortality risk associated with COVID‐19 in the current pandemic for the optimal management of the patients.^29^ Several inflammatory and hematological parameters are thought to associate with the severity of COVID‐19.^18^, ^24^ The current study was aimed to examine the different blood parameters in the confirmed positive COVID‐19 patients. We analyzed several blood parameters and their association with the severity of the COVID‐19 disease. In this study, we classified the patients into different categories based on disease severity. The patients were categorized into mild, moderate, severe, and critical groups. Among the total 101 patients, 52 were mild, 24 were moderate, 09 were severe and 16 were critical diseased patients. We recorded 16 deaths that were associated with the critical group.

Among the previously reported mortality predictors for COVID‐19, the increasing age was an important factor and associated with the poor outcomes.^12^, ^25^ The mean age for deceased individuals in our study was 62.12 ± 14.35 years which was greater than any other diseased group (represented in Table 4). The statistical analysis represented a significant association between the increased age and mortality due to COVID‐19. There was no association between the severity of COVID‐19 and gender in our study. Similar to our study, Hypertension, senility, diabetes, heart disease, COPD, and asthma were reported in different studies as predictors for severe COVID‐19.^30^, ^31^ A statistically significant difference was shown between comorbid conditions and mortality as shown in Table 2. A significant increase in the WBCs level in the critical and diseased individuals was observed. It has been previously reported that the WBCs count increased with the severity of the COVID‐19 disease.^26^ We observed a decrease in the lymphocytes in severe and critical patients. Based on our observation, it could be speculated that the lymphocytes count depletion is directly associated with the COVID‐19 disease severity and the survival rate of the disease could be linked with the ability of T lymphocytes which are essential for the destruction of infected viral particles.^32^ Our observation supports the previous investigations which documented differential diagnostic criteria for COVID‐19 patients based on the increased WBC count along with lymphopenia.^33^

**TABLE 4:** The frequency and mean age of COVID‐19‐positive patients in various diseased groups is represented

	Frequency	Mean age	*p* Value
Mild	52	43.24 ± 15.65	0.001
Moderate	24	49.17 ± 14.60	
Severe	9	56.66 ± 24.24	
Critical	16	62.12 ± 14.35	
Total	101	48.94	

Thrombocytopenia was increased with the severity of the disease and the lowest value of platelets was recorded for critical patients. Our findings are consistent with the previous studies in which thrombocytopenia was associated with severely diseased individuals and dead patients. Therefore, thrombocytopenia could be used as a useful indicator for disease progression.^33^ The mechanism of thrombocytopenia in COVID‐19 and other coronavirus diseases is still unknown which needs to be explored.^34^ The platelet count was decreased in the severe and critical patients which could be linked to thrombin generation, immunological destruction of platelets, impaired megakaryopoiesis, and inappropriate platelet consumption.^35^ Examination of the platelet could be a suitable biomarker for recognition of coagulopathy and its severity.

The lymphocytes functions testing has been suggested to assess the severity of COVID‐19 disease.^4^ We observed the decreased lymphocytes count and increased granulocytes in the critical diseased individuals which could be attributed to increased inflammation and suppression of the immune system caused by SARS‐CoV‐2 infection. The elevation in granulocytes and decrease in lymphocytes can be, therefore, easily used for severity and mortality analysis of COVID‐19 as routine blood tests are easy and readily available.^10^, ^34^ The lymphocytopenia has been reported to be common events in the previous coronavirus outbreaks that is, MERS and SARS.^36^ Our results are in accordance with the previous investigation where the lymphocytopenia was common in MERS‐CoV and SARS‐CoV infection.^35^ Various studies have supported lymphocytopenia as a reliable and effective biomarker for the severity of COVID‐19 disease.^36^, ^37^ Similarly, the statistical analysis for neutrophil‐to‐lymphocyte ratio revealed 2.444 median for all groups while the coefficient of dispersion was 1.001. The highest median was observed to be 5.851 for the critical group and the lowest (1.857) for the mild group. The statistical analysis for platelets to lymphocyte ratio revealed 123.333 median for all groups while the coefficient of dispersion was 0.616. The highest median was observed to be 1.63 for the moderate group and the lowest (105.606) for the mild group. A significant association was observed in Neutrophil‐to‐lymphocyte ratio (NLR), Lymphocyte‐to‐monocyte ratio (LMR), and Platelet‐to‐lymphocyte ratio (PLR) among various disease groups. As COVID‐19 causes a systemic inflammatory response, neutrophils are activated by virus‐induced inflammatory markers IL‐6 and IL‐8, GCSF, IFN‐ϒ, TNF‐α formed by lymphoid and endothelial cells. Conversely, the immune response is considerably depressed notably the helper T lymphocytes. Hence, NLR is elevated as a result and it is associated with disease progression. Previous investigations have also reported neutrophil to lymphocytes and platelets to lymphocytes ratio as important prognostic factors for disease progression.^10^, ^38^ Further, Sit et al^39^ have shown that raised NLR in patients with thyroid nodules in preoperative period serves as an indicator of underlying malignant nodular disease. Hence, this should encourage the physician to perform cancer screening in thyroid gland in such patients. Similarly, elevated PDW, MLR, and NLR in healthy individuals can also be associated with liver damage and steatosis and Non‐Alcoholic Fatty liver disease (NAFLD), proving the relationship between hepatic steatosis and inflammatory markers.^40^ Additionally, patients with severe COVID‐19 infection were reported to have a higher NLR than those with non‐severe COVID‐19 infection.^9^, ^41^, ^42^, ^43^

### 4.1 Limitations of the study

The present study has several limitations, particularly the very small sample size. Therefore, exact implication of our findings on a large scale population could not be a good practice. Importantly, this is the first study from Pakistan to the best of our knowledge and our findings could be very helpful for policy makers in the current pandemic. The cases of COVID‐19 are surging as the second wave has been started globally. We, therefore, believe that the current study could provide more important literature while dealing with the cases of COVID‐19 in the overburdened hospitals.^44^, ^45^ All the subjects in our study were Pakistani nationals and therefore our findings could hardly be inferred to other ethnic groups. Further, we only reported hospitalized patients in our study. Non‐hospitalized individuals, and their clinical characteristics were not studied. Further exploration of the topic is urgently needed to provide optimal management to the patients during the COVID‐19 pandemic.

## 5 CONCLUSION

Several blood parameters need to be continuously screened in the current pandemic to assess the severity and mortality of COVID‐19. Thes parameters such as lymphocytes, white blood cells, and platelet count could be used as suitable biomarkers for prognosis of COVID‐19 disease. The elevation in the mentioned parameters can be easily used for severity and mortality analysis of COVID‐19 as routine blood tests are easy and readily available. The topic needs to be explored further for the early target of treatment options and optimal management of the COVID‐19 patients during the pandemic. Evaluating novel hemograms NLR, PLR, and LMR can aid clinicians to identify potentially severe cases at early stages, initiate effective management in time, and conduct early triage which may reduce the overall mortality of COVID‐19 patients.

## CONFLICT OF INTEREST

The author has no competing interests.

## Data Availability

The authors confirm that the data supporting the findings of this study are available within the article.
